# Supplementary material for: Cancer-specific alterations in nuclear matrix proteins determined by multi-omics analyses of ductal carcinoma in situ
Source: Front Oncol. 2024 Aug 6;14:1406946. doi: 10.3389/fonc.2024.1406946 (PMC11333849; doi:10.3389/fonc.2024.1406946)
Supplement: Supplementary file 1 [file DataSheet_1.pdf]

Supplementary Table 1. Clinical characteristics of non-diseased and breast cancer PDCEEs used in this study.

PDCEE	**Tumor Stage**	**Molecular Subtype**
JL-BRL-6 (Breast Reduction mammoplasty PDCEE)		
JL-Contra-3 Contralateral Breast PDCEE for JL-DCIS-3		
JL-DCIS-3	Stage 0	Luminal A
JL-BTL-4	Stage I	Luminal B
JL-BTL-8	Stage I	Luminal A
JL-BTL-33	Stage I	Luminal A
JL-BTL-37	Stage I	Luminal A
JL-BTL-9	Stage II	Luminal A
JL-BTL-10	Stage II	TNBC
JL-BTL-29	Stage II	TNBC
JL-BTL-46	Stage II	Her2 overexpressing
JL-BTL-12	Stage III	Luminal A
JL-BTL-21	Stage IV	Luminal B like
JL-BTL-60	Stage IV	TNBC
MDA-MB231	Stage IV	TNBC
MCF-7	Stage IV	Luminal A

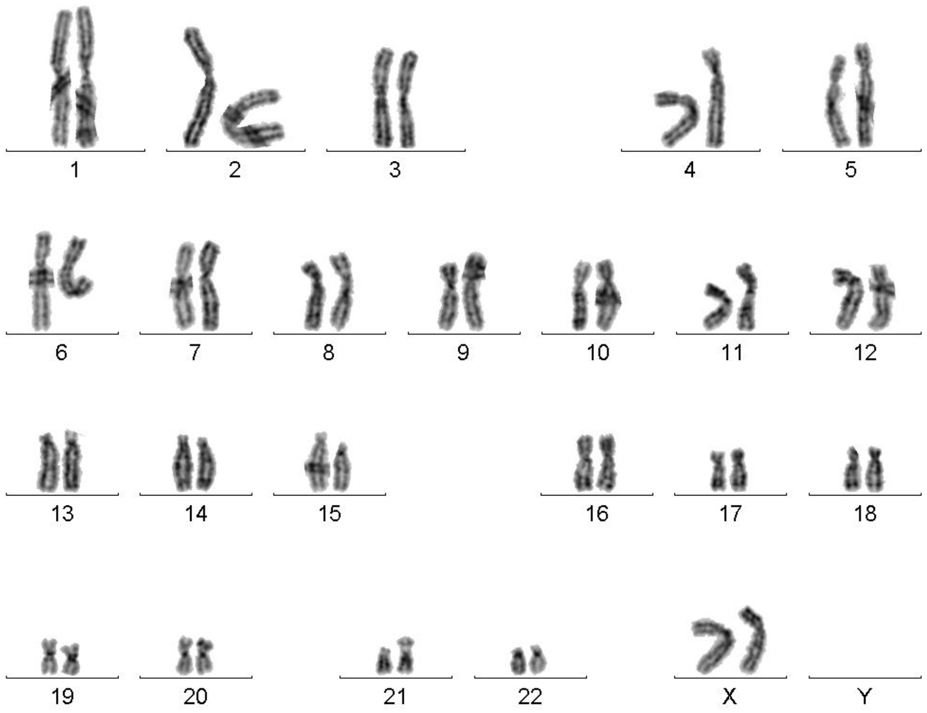

Supplementary Figure 1A. Karyotype of JL-Contra-3 PDCEE showing a diploid normal female chromosome complement.

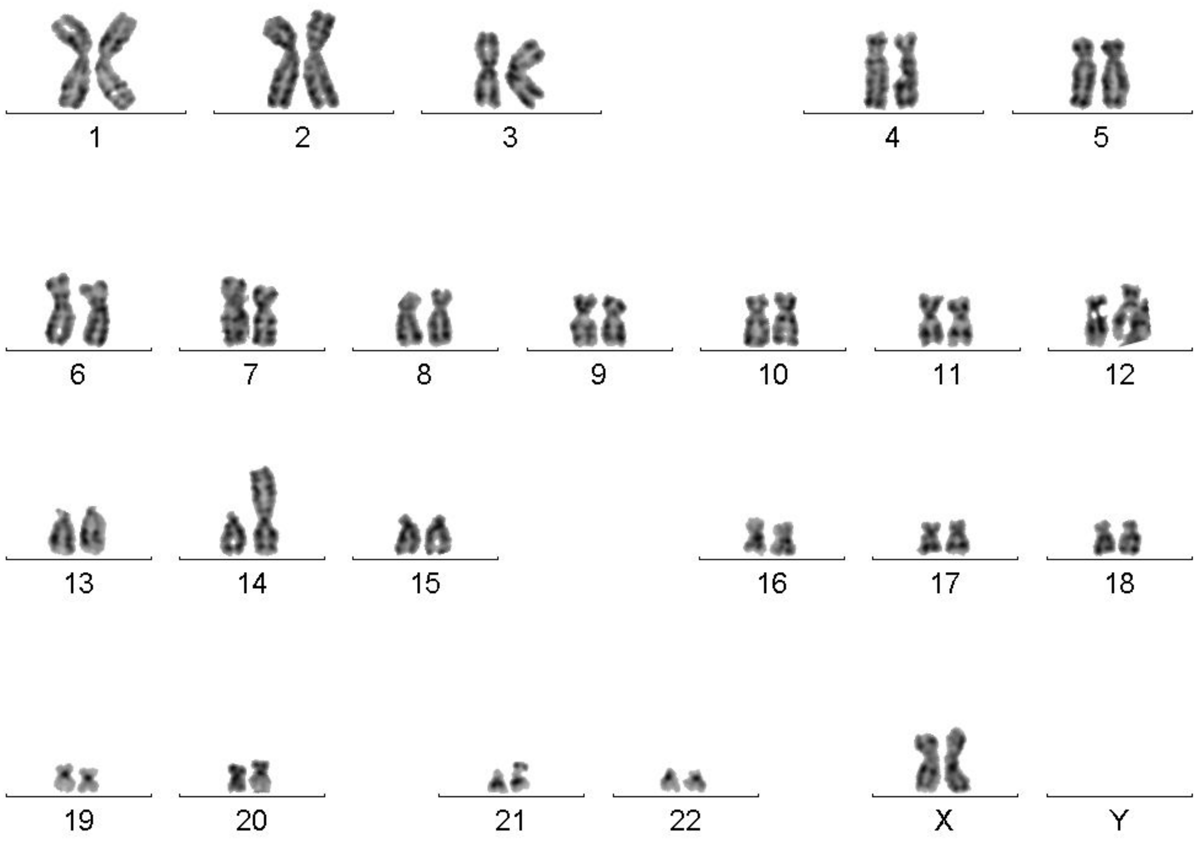

Supplementary Figure 1B. Karyotype of JL-DCIS-3 PDCEE showing a derivative chromosome 14 with an extra copy of 5q. This karyotype represents a prominent subclone of this patient-derived culture.

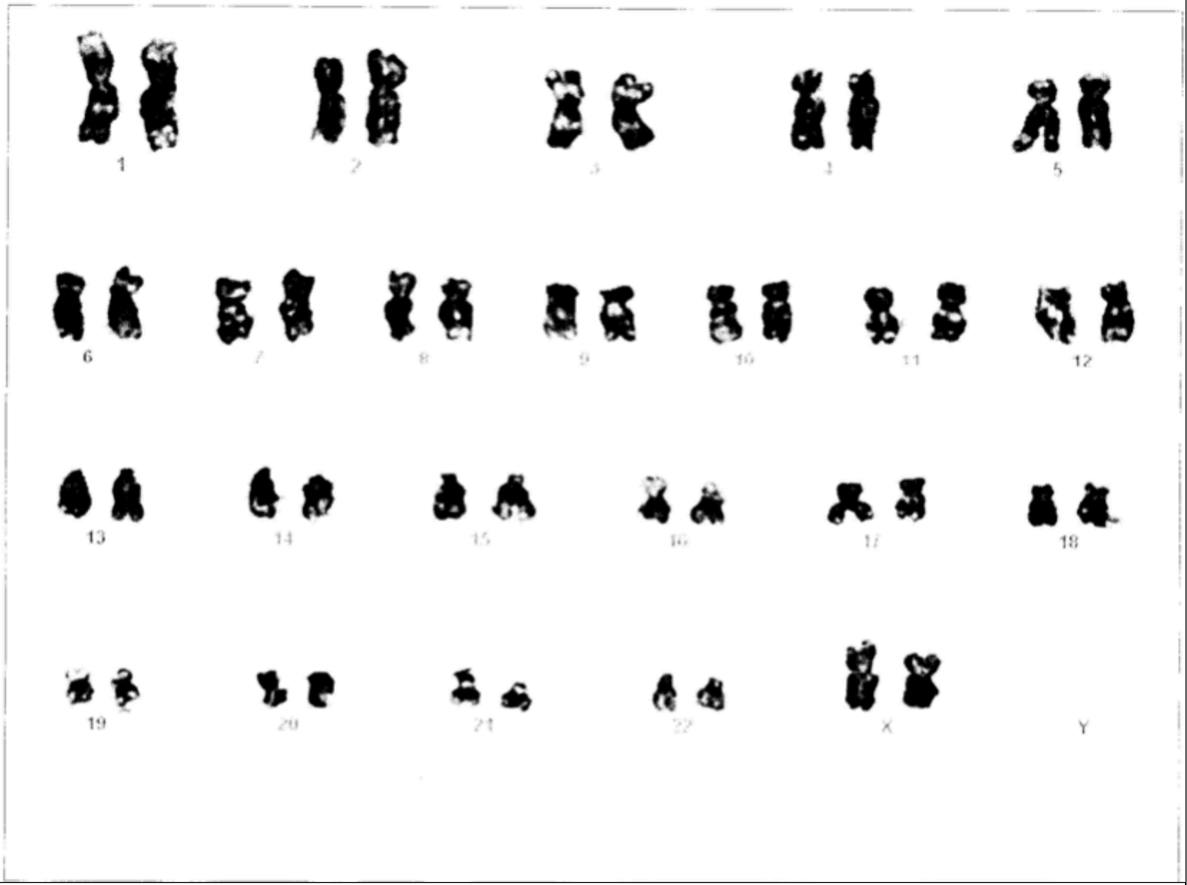

Supplementary Figure 1C. Karyotype of JL-BRL-6 PDCEE showing a diploid normal female chromosome complement.

Supplementary Table 2. NMPs Identified by Proteomics

	Protein Name	Protein Symbol	Fold change JL-DCIS-3 relative to JL-Contra-3	P value
1.	Vimentin	VIM	1.69 (↓)	0.000
2.	40S ribosomal protein S3	RPS3	1.07 (↓)	0.846
3.	High mobility group protein B3	HMGB3	1.42 (↓)	0.597
4.	40S ribosomal protein S7	RPS7	1.81 (↓)	0.007
5.	Neuroblast differentiation-associated protein AHNAK	AHNAK	5.35 (↓)	0.000
6.	Nucleolin	NCL	3.65 (↓)	0.000
7.	Keratin, type II cytoskeletal 8	KRT8	1.45 (↓)	0.038
8.	Isoform B1 of hnRNP A2/B1	HNRNPA2B1	1.78 (↓)	0.111
9.	Isoform 1 of Nucleophosmin	NPM1	4.98 (↓)	0.000
10.	Lamin-B2	LMNB2	1.19 (↓)	0.207
11.	Histone H1.2	HIST1H1C	2.38 (↓)	0.710
12.	Putative elongation factor 1-alpha-like	EEF1A1	1.84 (↓)	0.155
13.	Myosin regulatory light chain MRLC2	MYL12B	1.05 (↓)	0.059
14.	60 kDa heat shock protein	HSPD1	1.21 (↓)	0.338
15.	High mobility group protein B1	HMGB1	4.51 (↓)	0.039
16.	Putative uncharacterized protein DKFZp564G0422	ATPIF1	1.16 (↓)	0.723
17.	Isoform 1 of Myosin-9	MYH9	1.01 (↓)	0.958
18.	Activated RNA polymerase II transcriptional coactivator p15	SUB1	3.01 (↓)	0.188
19.	cDNA FLJ56329, highly similar to myosin light polypeptide 6	MYL6B	1.36 (↓)	0.393
20.	Putative uncharacterized protein MSN (Fragment)	MSN	2.46 (↓)	0.103
21.	Transcription factor A, mitochondrial	TFAM	1.62 (↓)	0.143
22.	Isoform A1-A of hnRNP A1	HNRNPA1	6.42 (↓)	0.008
23.	Isoform 1 of Heat shock cognate 71 kDa protein	HSPA8	1.17 (↓)	0.617
24.	Eukaryotic translation elongation factor 1 delta isoform 3	EEF1D	1.27 (↓)	0.504
25.	Stress-70 protein, mitochondrial	HSPA9	1.03 (↓)	0.906
26.	Isoform long of splicing factor, proline- and glutamine-rich	SFPQ	2.34 (↓)	0.000
27.	Isoform 1 of hnRNP H3	HNRNPH3	1.25 (↓)	0.233
28.	51 kDa protein	HNRNPH1	3.73 (↓)	0.041
29.	hnRNP F	HNRNPF	1.31 (↓)	0.403

30.	Putative uncharacterized protein hnRNP AB	HNRNPAB	2.59 (↓)	0.002
31.	Isoform 3 of clathrin light chain A	CLTA	1.18 (↓)	0.712
32.	Keratin 7	KRT7	1.43 (↓)	0.134
33.	Elongation factor 1-beta	EEF1B2	3.03 (↓)	0.002
34.	Isoform 1 of hnRNP Q	SYNCRIP Q	1.78 (↓)	0.413
35.	Isoform 1 of protein disulfide-isomerase A6	PDIA6	1.18 (↓)	0.575
36.	Protein disulfide-isomerase A3	PDIA3	1.30 (↓)	0.095
37.	Putative uncharacterized protein SPTAN1	SPTAN1	1.99 (↓)	0.000
38.	Filamin B	FLNB	1.14 (↓)	0.755
39.	Peroxiredoxin-1	PRDX1	1.29 (↓)	0.237
40.	Isoform 2 of hnRNP M	HNRNPM	1.54 (↓)	0.034
41.	Hepatoma-derived growth factor	HDGF	1.79 (↓)	0.285
42.	Endoplasmin	HSP90B1	2.29 (↓)	0.005
43.	Peptidyl-prolyl cis-trans isomerase B	PPIB	1.62 (↓)	0.238
44.	Transitional endoplasmic reticulum ATPase	VCP	1.51 (↓)	0.191
45.	High mobility group protein B2	HMGB2	4.51 (↓)	0.039
46.	DnaJ homolog subfamily B member 11	DNAJB11	1.45 (↓)	0.531
47.	45 kDa protein	ALDOA	1.73 (↓)	0.736
48.	Histone H1.4	HIST1H1E	5.57 (↓)	0.023
49.	THO complex subunit 4	THOC4	4.78 (↓)	0.043
50.	HSPA1A cDNA FLJ54392, highly similar to heat shock 70 kDa protein 1	HSPA1B	1.09 (↓)	0.882
51.	Non-POU domain-containing octamer-binding protein	NONO	2.31 (↓)	0.002
52.	Enhancer of rudimentary homolog	ERH	2.71 (↓)	0.061
53.	Putative uncharacterized protein hnRNP D	HNRNPD	1.25 (↓)	0.472
54.	Fus-like protein (Fragment)	FUS	4.66 (↓)	0.220
55.	18 kDa protein	Ribosomal 23	1.58 (↓)	0.055
56.	cDNA FLJ35087 fis, clone PLACE6005546, highly similar to polymerase I and transcript release factor	PTRF	2.12 (↓)	0.151
57.	Stathmin 1 isoform b	STMN1	5.06 (↓)	0.024

58.	Ribosomal protein S21	RPS21	2.12 (↓)	0.0419
59.	cDNA FLJ31776 fis, clone NT2RI2008141, highly similar to Calumenin	CALU	3.39 (↓)	0.068
60.	Chromobox protein homolog 3	CBX3	2.70 (↓)	0.047
61.	Sin3A-associated protein, 18kDa	SAP18	1.13 (↓)	0.823
62.	NADH-ubiquinone oxidoreductase flavoprotein 3 isoform a precursor	NDUFV3	1.069 (↓)	0.899
63.	18 kDa protein	UBC	1.80 (↓)	0.312
64.	Isoform 2 of polyadenylate-binding protein 2	PABPN1	4.20 (↓)	0.041
65.	Isoform brain of clathrin light chain B	CLTB	2.30 (↓)	0.005
66.	Isoform 3 of hnRNP K	HNRNPK	2.20 (↓)	0.034
67.	Splicing factor, arginine/serine-rich 9	SFRS9	1.48 (↓)	0.403
68.	highly similar to 40S ribosomal protein S20	RPS20	3.06 (↓)	0.173
69.	LIM domain and actin binding 1 isoform a	LIMA1	4.16 (↓)	0.023
70.	Alpha-enolase	ENO1	1.01 (↓)	0.979
71.	Nucleoprotein TPR	TPR	1.14 (↓)	0.686
72.	FK506-binding protein 3	FKBP3	1.91 (↓)	0.272
73.	Isoform 1 of hnRNP D-like	HNRPDL	8.41 (↓)	0.015
74.	Elongation factor 2	EEF2	1.44 (↓)	0.193
75.	hnRNP G	RBMX	1.90 (↓)	0.037
76.	Isoform 2 of hnRNP A3	HNRNPA3	2.13 (↓)	0.001
77.	Isoform HMG-Y of High mobility group protein HMG-I/HMG-Y	HMGA1	4.03 (↓)	0.006
78.	69 kDa protein	EZR	4.59 (↓)	0.000
79.	High mobility group protein B3-like-1	HMGB3L1	1.42 (↓)	0.597
80.	Isoform 2 of ATP synthase subunit d, mitochondrial	ATP5H	1.39 (↓)	0.441
81.	19 kDa protein	LASP1	1.72 (↓)	0.277
82.	Small nuclear ribonucleoprotein D2	SNRPD2	1.91 (↓)	0.361
83.	Prefoldin subunit 4	PFDN4	1.38 (↓)	0.430
84.	Eukaryotic translation initiation factor 3 subunit I	EIF3I	1.20 (↓)	0.688
85.	hnRNP R isoform 1	HNRNPR	1.88 (↓)	0.154

86.	DnaJ homolog subfamily A member 1	DNAJA1	1.19 (↓)	0.247
87.	Stomatin-like protein 2	STOML2	1.01 (↓)	0.989
88.	PARP1 Poly [ADP-ribose] polymerase 1	PARP1	1.10 (↓)	0.754
89.	Talin-1	TLN1	1.86 (↓)	0.112
90.	Isoform 1 of Tropomyosin alpha-4 chain	TPM4	2.45 (↓)	0.031
91.	Heterogeneous nuclear ribonucleoprotein L	HNRNPL	1.55 (↓)	0.349
92.	CAST Isoform 6 of Calpastatin	CAST	6.74 (↓)	0.012
93.	40S ribosomal protein S17	RPS17	1.81 (↓)	0.281
94.	Zinc finger protein 185	ZNF185	5.07 (↓)	0.033
95.	Histone H1.0	H1F0	1.02 (↓)	0.898
96.	CDC37 Hsp90 co-chaperone Cdc37	CDC37	9.36 (↓)	0.043
97.	Staufen isoform c	STAU1	1.41 (↓)	0.257
98.	Stress-induced-phosphoprotein 1	STIP1	1.33 (↓)	0.701
99.	Eukaryotic translation initiation factor 3 subunit J	EIF3J	1.95 (↓)	0.054
100.	Isoform 2 of A-kinase anchor protein 2	AKAP2	3.27 (↓)	0.075
101.	EIF1AX;EIF1AP1 Eukaryotic translation initiation factor 1A, X-chromosomal	EIF1AX	4.75 (↓)	0.345
102.	CCT2 T-complex protein 1 subunit beta	CCT2	1.67 (↓)	0.589
103.	Coiled-coil domain-containing protein 124	CCDC124	2.62 (↓)	0.155
104.	Isoform 2 of Coiled-coil domain-containing protein 50	CCDC50	4.39 (↓)	0.237
105.	Isoform Long of TATA-binding protein-associated factor 2N	TAF15	3.59 (↓)	0.234
106.	RPS3A 40S ribosomal protein S3a	RPS3A	1.01 (↓)	0.992
107.	RPSAP15;RPSA 33 kDa protein	RPSAP15	1.821 (↓)	0.702
108.	Isoform 1 of Paraspeckle component 1	PSPC1	2.11 (↓)	0.066
109.	Vasodilator-stimulated phosphoprotein	VASP	1.80 (↓)	0.427
110.	Nuclear migration protein nudC	NUDC	2.84 (↓)	0.020
111.	Isoform 3 of Spectrin alpha chain, brain	SPTBN1	1.99 (↓)	0.000

112.	RPLP2 60S acidic ribosomal protein P2	RPLP2	2.00 (↓)	0.382
113.	Isoform 2 of Splicing factor U2AF 65 kDa subunit	U2AF2	1.95 (↓)	0.082
114.	Keratin, type I cytoskeletal 9	KRT9	1.02 (↓)	0.976
115.	Serine/threonine-protein kinase 10	STK10	1.10 (↓)	0.722
116.	Isoform 1 of Adenylyl cyclase-associated protein 1	CAP1	3.52 (↓)	0.304
117.	SWI/SNF-related matrix-associated actin-dependent regulator of chromatin subfamily A member 5	SMARCA5	1.04 (↓)	0.841
118.	cDNA FLJ56016, highly similar to C-1-tetrahydrofolate synthase	MTHFD1	1.69 (↓)	0.184
119.	Protein BUD31 homolog	BUD31	2.52 (↓)	0.004
120.	Isoform 1 of Kinectin	KTN1	1.16 (↓)	0.799
121.	Tyrosyl-tRNA synthetase	YARS	1.21 (↓)	0.806
122.	Radixin, isoform CRA_a	RDX	1.42 (↓)	0.703
123.	Isoform 6 of Microtubule-associated protein 4	MAP4	1.72 (↓)	0.772
124.	Tropomodulin-3	TMOD3	1.82 (↓)	0.301
125.	T-complex protein 1 subunit zeta	CCT6A	2.36 (↓)	0.126
126.	Isoform 1 of RuvB-like 1	RUVBL1	1.86 (↓)	0.355
127.	-14 kDa protein		1.28 (↓)	0.705
128.	-19 kDa protein		1.34 (↓)	0.808
129.	-21 kDa protein	CD99	2.17 (↓)	0.139
130.	Stress-induced-phosphoprotein 1	STIP1	1.33 (↓)	0.701
131.	Cell division cycle 5-like protein	CDC5L	1.73 (↓)	0.290
132.	Cytochrome c oxidase subunit 5A, mitochondrial	COX5A	1.36 (↓)	0.654
133.	Isoform Long of Eukaryotic translation initiation factor 4H	EIF4H	1.45 (↓)	0.490
134.	Putative uncharacterized protein MATR3	MATR3	1.31 (↓)	0.652
135.	Isoform Long of Antigen KI-67	MKI67	4.01 (↓)	0.461
136.	RPS6 40S ribosomal protein S6	RPS6	1.15 (↓)	0.622
137.	SNRPB Putative uncharacterized protein SNRPB	SNRPB	1.13 (↓)	0.787
138.	Thioredoxin domain-containing protein 5	TXNDC5	1.16 (↓)	0.553
139.	EIF3G Eukaryotic translation initiation factor 3 subunit G	EIF3G	2.16 (↓)	0.147

140.	Isoform 1 of Caprin-1	CAPRIN1	1.88 (↓)	0.401
141.	Nucleolar protein 16	NOP16	3.09 (↓)	0.182
142.	Isoform 1 of Transcription intermediary factor 1-beta	TRIM28	1.41 (↓)	0.736
143.	BCAS2 Pre-mRNA-splicing factor SPF27	BCAS2	1.15 (↓)	0.122
144.	SEPT7 51 kDa protein	SEPT7	1.45 (↓)	0.078
145.	HDLBP Vigilin	HDLBP	1.04 (↓)	0.937
146.	Isoform 2 of Drebrin	DBN1	1.40 (↓)	0.364
147.	DNAJB1 DnaJ homolog subfamily B member 1	DNAJB1	4.05 (↓)	0.005
148.	Actinin, alpha 1 isoform a	ACTN1	2.97 (↓)	0.459
149.	Isoform 2 of Vinculin	VCL	1.05 (↓)	0.873
150.	TPM1 37 kDa protein	TPM1	4.33 (↓)	0.012
151.	PRDX3 peroxiredoxin 3 isoform b	PRDX3	2.22 (↓)	0.135
152.	Isoform 3 of Splicing factor 1	SF1	1.31 (↓)	0.707
153.	CCT5 T-complex protein 1 subunit epsilon	CCT5	1.52 (↓)	0.572
154.	SNRPD3 Putative uncharacterized protein SNRPD3	SNRPD3	1.61 (↓)	0.027
155.	SRP14 Signal recognition particle 14 kDa protein	SRP14	1.05 (↓)	0.915
156.	DNAJA2 DnaJ homolog subfamily A member 2	DNAJA2	2.38 (↓)	0.173
157.	-52 kDa protein		1.00	1.000
158.	60S ribosomal protein L14	RPL14 6	-	
159.	Keratin, type I cytoskeletal 18	KRT18	1.41 (↑)	0.160
160.	Keratin, type I cytoskeletal 19	KRT19	1.01 (↑)	0.988
161.	Filamin A, alpha	FLNA	1.55 (↑)	0.054
162.	Isoform 3 of plectin-1	PLEC1	1.94 (↑)	0.000
163.	ATP synthase subunit alpha, mitochondrial	ATP5A1	2.02 (↑)	0.039
164.	60S ribosomal protein L8	RPL8	5.46 (↑)	0.002
165.	Isoform ASF-1 of splicing factor, arginine/serine-rich 1	SFRS1	1.10 (↑)	0.718
166.	Tubulin beta chain	TUBB	2.26 (↑)	0.408
167.	Isoform 2 of polyadenylate-binding protein 1	PABPC1	1.04 (↑)	0.873
168.	Laminin subunit gamma-1	LAMC1	9.87 (↑)	0.038
169.	Isoform 3 of plasminogen activator inhibitor 1 RNA-binding protein	SERBP1	3.58 (↑)	0.303

170.	Glyceraldehyde-3-phosphate dehydrogenase	GAPDH	3.53 (↑)	0.004
171.	Heat shock protein beta-1	HSPB1	2.06 (↑)	0.023
172.	Putative uncharacterized protein ALB	ALB	6.94 (↑)	0.003
173.	28 kDa heat- and acid-stable phosphoprotein	PDAP1	2.40 (↑)	0.037
174.	40S ribosomal protein S23	RPS23	2.20 (↑)	0.111
175.	Isoform 1 of triosephosphate isomerase	TPI1	2.27 (↑)	0.225
176.	Annexin A1	ANXA1	2.29 (↑)	0.014
177.	40S ribosomal protein S19	RPS19	1.13 (↑)	0.232
178.	Tu translation elongation factor	TUFM	1.78 (↑)	0.082
179.	Keratin, type II cytoskeletal 1	KRT1	2.96 (↑)	0.011
180.	Splicing factor, arginine/serine-rich 3, isoform CRA_a	SFRS3	1.33 (↑)	0.001
181.	Putative uncharacterized protein MECP2 (Fragment)	MECP2	4.66 (↑)	0.002
182.	Isoform 2 of DNA-binding protein A	CSDA	4.60 (↑)	0.059
183.	Similar to ribosomal protein L23	RPL23	1.59 (↑)	0.168
184.	heat shock 90kDa protein 1, alpha isoform 1	HSP90AA1	1.16 (↑)	0.692
185.	Isoform UBF2 of nucleolar transcription factor 1	UBTF	1.36 (↑)	0.424
186.	Transcription factor BTF3 homolog 4	BTF3L4	2.82 (↑)	0.172
187.	40S ribosomal protein S4, X isoform	RPS4X	2.39 (↑)	0.353
188.	Charged multivesicular body protein 4b	CHMP4B	1.33 (↑)	0.074
189.	Isoform 1 of RNA-binding protein raly	RALY	1.21 (↑)	0.464
190.	Isoform 2 of 60S ribosomal protein L11	RPL11	3.66 (↑)	0.021
191.	Protein disulfide-isomerase	P4HB P	3.40 (↑)	0.068
192.	60S ribosomal protein L7a	RPL7A	3.95 (↑)	0.049
193.	PKM2 cDNA FLJ56065, highly similar to pyruvate kinase isozyme M1	PKM2	2.59 (↑)	0.097
194.	27 kDa protein	CHCHD3	1.09 (↑)	0.825
195.	Isoform 1 of caldesmon	CALD1	1.50 (↑)	0.112
196.	Laminin subunit beta-1	LAMB1	1.30 (↑)	0.396
197.	Histone H4	HIST1H4D	1.11 (↑)	0.618

198.	Isoform 1 of 60S ribosomal protein L12	RPL12	1.38 (↑)	0.730
199.	Putative uncharacterized protein DKFZp686J1372	TPM3	1.01 (↑)	0.971
200.	Peroxiredoxin-2	PRDX2	1.09 (↑)	0.845
201.	Isoform 1 of cytoskeleton-associated protein 4	CKAP4 I	7.55 (↑)	0.000
202.	Serpin H1	SERPINH1	1.26 (↑)	0.684
203.	HSPA5 protein	HSPA5	1.04 (↑)	0.846
204.	Annexin A2 isoform 1	ANXA2	1.07 (↑)	0.685
205.	p180/ribosome receptor	RRBP1	1.55 (↑)	0.089
206.	26S protease regulatory subunit 6A	PSMC3	1.08 (↑)	0.753
207.	40S ribosomal protein S14	RPS14	1.65 (↑)	0.118
208.	Nuclease-sensitive element-binding protein 1	YBX1	1.07 (↑)	0.860
209.	Cytochrome c oxidase subunit 5B	COX5B	2.45 (↑)	0.296
210.	interleukin enhancer binding factor 3 isoform d	ILF3	1.47 (↑)	0.237
211.	Isoform long of hnRNP U	HNRNPU	1.01 (↑)	0.982
212.	10 kDa heat shock protein	HSPE1	1.01 (↑)	0.995
213.	Putative uncharacterized protein RPL31	RPL31	4.00 (↑)	0.018
214.	cDNA FLJ59206, highly similar to Eukaryotic translation initiation factor 4B	FLJ59206	1.25 (↑)	0.771
215.	60S ribosomal protein L3	RPL3	1.25 (↑)	0.794
216.	Isoform 2 of Na(+)/H(+) exchange regulatory cofactor NHE-RF2	SLC9A3R2	2.25 (↑)	0.417
217.	Dihydrolipoyllysine-residue succinyltransferase component of 2-oxoglutarate dehydrogenase complex, mitochondrial	DLST	2.18 (↑)	0.193
218.	Nascent polypeptide-associated complex subunit alpha-2	NACA	2.10 (↑)	0.084
219.	Isoform 2 of nucleoside diphosphate kinase A	NME1	1.98 (↑)	0.122
220.	Similar to hCG1812668	RPL26P14	4.64 (↑)	0.196
221.	hnRNP H2	HNRNPH2	1.25 (↑)	0.580
222.	Heat shock protein HSP 90-beta	HSP90AB1	1.48 (↑)	0.486
223.	60S ribosomal protein L22	RPL22	1.66 (↑)	0.413

224.	Similar to 60S ribosomal protein L17	Ribosomal L22	1.66 (↑)	0.413
225.	Phosphoglycerate kinase 1	PGK1	2.77 (↑)	0.080
226.	cDNA FLJ60076, highly similar to ELAV-like protein 1	ELAVL1	1.10 (↑)	0.805
227.	Topoisomerase (DNA) I	TOP1	1.94 (↑)	0.340
228.	Isoform 1 of apoptotic chromatin condensation inducer in the nucleus	ACIN1	2.078 (↑)	0.291
229.	Highly similar to probable ATP-dependent RNA helicase DDX5	DDX5	1.47 (↑)	0.026
230.	Putative uncharacterized protein WIBG (Fragment)	WIBG	2.12 (↑)	0.124
231.	Ribosomal protein S8	RPS8	1.01 (↑)	0.998
232.	Kinesin-1 heavy chain	KIF5B	1.01 (↑)	0.970
233.	Prohibitin variant (fragment)	PHB	1.49 (↑)	0.109
234.	cDNA FLJ59206, highly similar to Eukaryotic translation initiation factor 4B	EIF4B	1.25 (↑)	0.771
235.	RBM4 Isoform 1 of RNA-binding protein 4	RBM4	1.11 (↑)	0.472
236.	HIST1H2BF; histone cluster 1, H2bg	HIST1H2BF	1.22 (↑)	0.507
237.	NME2; NME1 Nucleoside diphosphate kinase	NME2	1.98 (↑)	0.122
238.	Putative uncharacterized protein IMMT	IMMT	1.40 (↑)	0.516
239.	Isoform 1 of High mobility group protein 20A	HMG20A	1.49 (↑)	0.555
240.	Isoform 2 of LIM domain only protein 7	LMO7	2.70 (↑)	0.326
241.	Heterogeneous nuclear ribonucleoprotein H2	RPL36AP37	1.25 (↑)	0.580
242.	Isoform 1 of Splicing factor, arginine/serine-rich 7	SFRS7	2.96 (↑)	0.012
243.	Tubulin-specific chaperone A	TBCA	3.54 (↑)	0.234
244.	Dihydrolipoyllysine-residue succinyltransferase component of 2-oxoglutarate dehydrogenase complex, mitochondrial	DLSTP	2.18 (↑)	0.193
245.	Lon protease homolog	LONP1	1.62 (↑)	0.416
246.	TKT Transketolase	TKT	3.63 (↑)	0.285

247.	Basement membrane-specific heparan sulfate proteoglycan core protein variant	HSPG2	3.49 (↑)	0.137
248.	UMP-CMP kinase 1 isoform a	CMPK1	1.53 (↑)	0.658
249.	Putative uncharacterized protein CTNNA1	CTNNA1	1.64 (↑)	0.375
250.	ACIN1 apoptotic chromatin condensation inducer 1 isoform 3	ACIN1	2.07 (↑)	0.291
251.	Alpha-actinin-4	ACTN4	1.35 (↑)	0.312
252.	28 kDa protein	ARHGDIA	1.68 (↑)	0.703
253.	ATP5O ATP synthase subunit O, mitochondrial	ATP5O	1.14 (↑)	0.836
254.	Cofilin-1	CFL1	1.65 (↑)	0.359
255.	Isoform DPI of Desmoplakin	DSP	2.15 (↑)	0.003
256.	Eukaryotic translation initiation factor 3 subunit A	EIF3A	1.23 (↑)	0.498
257.	Isoform 2 of Electron transfer flavoprotein subunit beta	ETFB	1.23 (↑)	0.665
258.	Putative rRNA methyltransferase 3	FTSJ3	1.14 (↑)	0.817
259.	Histone H4	HIST1H4K	1.11 (↑)	0.618
260.	Isoform 1 of Heterochromatin protein 1-binding protein 3	HP1BP3	1.10 (↑)	0.727
261.	Prefoldin subunit 2	PFDN2	1.02 (↑)	0.962
262.	RPS18P9; RPS18 40S ribosomal protein S18	RPS18	2.08 (↑)	0.269
263.	FUBP1 Putative uncharacterized protein FUBP1	FUBP1	1.69 (↑)	0.291
264.	Tubulin alpha-1B chain	TUBA1B	5.18 (↑)	0.198
265.	PDLIM5 PDZ and LIM domain protein 5	PDLIM5	2.33 (↑)	0.280
266.	Isoform 2 of Polypyrimidine tract-binding protein 1	PTBP1	1.95 (↑)	0.688
267.	PRSS1 28 kDa protein	PRSS1	2.15 (↑)	0.0483
268.	Programmed cell death protein 5	PDCD5	1.27 (↑)	0.833
269.	RPS28 40S ribosomal protein S28	RPS28	1.22 (↑)	0.687
270.	Peptidyl-prolyl cis-trans isomerase A	PPIA	1.70 (↑)	0.265
